# Consistency of the estimated target weights and ECW/TBW using BIA after hemodialysis in patients between standing and lying-down positions

**DOI:** 10.1186/s12882-022-02737-3

**Published:** 2022-03-17

**Authors:** Gwangho Choi, Ho Joong Yoon, Young Jin Song, Hae Min Jeong, Jae Eon Gu, Miyeun Han, Seok Hyung Kim, Jong-Woo Yoon, Hyunsuk Kim

**Affiliations:** 1 Department of Internal Medicine, Hallym University Medical Center, Chuncheon Sacred Heart Hospital, Chuncheon-si, Gangwon-do 24253 Republic of Korea; 2 Department of Internal Medicine, Hallym University Medical Center, Hangang Sacred Heart Hospital, Seoul, 07247 Republic of Korea

**Keywords:** Hemodialysis, Bioimpedance analysis, Estimated target weight

## Abstract

**Background:**

As hemodialysis is administered with the patient lying down, the distribution of body fluid is stable in the lying position, which is why this position is recommended for bioimpedance analysis (BIA). Although the InBody S10 is widely used for hemodialysis patients in the lying position, clinicians must make the measurements in person. In contrast, patients can use the InBody 770 to obtain measurements by themselves in the standing position, which may be more convenient. Therefore, this study compared the measurements of hemodialysis patients’ estimated target weight and ECW/TBW obtained lying down using the S10 to those obtained in the standing position using the 770.

**Methods:**

This study was conducted among maintenance hemodialysis patients at Chuncheon Sacred Heart Hospital in October 2020. Measurements from 56 patients before and after hemodialysis were obtained using the 2 machines. Each (S10 or 770) estimated target weight, both pre- and post-hemodialysis, was considered ideal when the ECW/TBW ratio was 0.380. R^2^ was calculated and the Bland-Altman test was performed.

**Results:**

The patients’ median age was 64 years old, and 51% were men. The actual ultrafiltration was 2 kg, and the mean TBW change measured using the InBody devices was 1.5 L (*R*^2^ = 0.718) for the S10 and 1.7 L (*R*^2^ = 0.616) for the 770. The estimated target weight at pre- and post-hemodialysis showed a remarkably high correlation with the patients’ actual pre- and post-hemodialysis weight (*R*^2^ > 0.095). The correlation between these measurements (lying vs. standing) before and after hemodialysis was also very close (*R*^2^ = 1.0000). In addition, ECW/TBW had a good correlation (R2 ≥ 0.970) The Bland-Altman test of dry weight and ECW/TBW yielded similar results.

**Conclusions:**

This study showed that patients’ estimated target weights in the lying position using the InBody S10 device and in the standing position using the InBody 770 device were consistent in both pre- and post-hemodialysis states.

**Supplementary Information:**

The online version contains supplementary material available at 10.1186/s12882-022-02737-3.

## Introduction

Bioimpedance analysis (BIA) is widely used to estimate the dry weight of hemodialysis (HD) patients. BIA is a noninvasive and simple technique that sends a weak electrical current through the body and calculates the impedance to measure the intracellular water (ICW), muscle mass, and fat with high accuracy and reproducibility. It has recently been used in a wide range of fields for the diagnosis of edema, obesity, and metabolic syndrome, as well as kidney disease and the evaluation of nutritional status and rehabilitation [1–5]. Since overhydration, as measured using BIA, is known to affect morbidity and mortality in patients [6, 7], the dry weight estimated using the ratio of extracellular water (ECW) to total body water (TBW) after HD can be useful data for clinicians [8, 9]. In addition, as the number of dialysis patients with diabetic kidney failure with replacement therapy, old age, and cardiovascular disease is increasing in recent years, it is important to optimize hydration for individual patients.

In general, since HD is administered with patients lying down, the distribution of their body fluid remains stable in the lying position, for which reason the lying position is recommended after HD. The InBody S10 is widely used for HD patients in the lying position [8, 10, 11]; however, a clinician must take the patient’s measurements in person in order to properly attach each electrode to the patient. Therefore, the disadvantage of the S10 is that it requires health-care personnel to perform the measurements. In contrast, patients can use the InBody 770 to obtain measurements by themselves in the standing position according to instructions provided by the machine [12], which can be more convenient and efficient for taking BIA measurements since there is no requirement for a clinician to be present. However, manufacturers do not typically recommend performing BIA measurements with the patient in the standing position due to theoretical body water imbalance caused by fluid movement after HD, which lasts for 4 h. If there is no significant difference between the BIA values measured using the S10 while lying down and the BIA values measured using the 770 while standing, then the 770 will likely be preferred due to its convenience when taking measurements and because it does not require the help of a clinician. However, no studies have investigated the degree of agreement or correlation between BIA measurements taken in the standing position with the 770 and in the lying position with the S10 in dialysis patients. The bioelectrical impedance analysis devices (InBody S10 and InBody 770) used in this study are based on multi-frequency BIA technology and use a 4-pole, 8-point detachable electrode method to measure impedance in five locations (right arm, left arm, torso, right leg, and left leg) at six frequencies (1, 5, 50, 250, 500, 1000 kHz). The 770 is an analytical device that matches the precision of the S10. Although its precision is acceptable for the general population [6] and individuals with fluid overload [13–15], research is needed to determine its level of precision according to posture in dialysis patients.

Therefore, this study aimed to estimate the dry weight and ECW/TBW in HD patients by examining the level of agreement between BIA values obtained using the 770 in the standing position before and after dialysis and the reference values obtained using the S10 in the lying down position.

## Methods

### Subjects and measurements

This study was conducted among maintenance HD patients who received treatment three times per week at Chuncheon Sacred Heart Hospital in October 2020. Only patients older than 18 who underwent BIA before and after HD were enrolled. Patients who had an acute illness within the previous three months, active cancer, pulmonary edema, liver cirrhosis with ascites, and class III or class IV congestive heart failure (using the New York Heart Association classification system), as well as those who experienced amputation or had lymphedema of the limbs, were excluded. Clinical information, including past medical history, the cause of kidney failure with replacement therapy [16], and HD vintage, was collected from the patients’ medical charts. BIA was measured during mid-week dialysis sessions (Wednesday or Thursday). Since the standing position should be maintained for about 2 min before taking measurements using the 770, only those who could grip the arm holders provided by the 770 with both hands and stand without assistance were enrolled.

In earlier study, we tested the correlations of resistances in the standing position of S10 and 770. Two machines (S10 and 770) were tested in a standing position for a general population (: *n* = 81 (M = 55 F = 26). The correlation between the two machines was very high (R2 ≥ 0.985, Supplementary Table 1 and supplementary Fig. 1).

Patients were instructed to consume their meals two hours before dialysis to prevent interference from digestion, and they were not permitted to eat during dialysis. Once patients arrived at the dialysis unit, they were instructed to stand for 5 min and BIA was measured using the 770 device (InBody, Seoul, South Korea) in an upright position. Then, patients rested for 10 min in lying position, after which BIA was measured using an S10 device (InBody, Seoul, South Korea). Electrodes were attached to both hands and legs. The electrodes on the hand were attached to the thumb and the middle finger, while the electrodes on the foot were attached on the inside on the medial side and on the outside of the lateral side. After the completion of pre-HD BIA measurements, blood tests were conducted simultaneously via venipuncture, and dialysis was then performed with the patient in the lying position. Once dialysis was completed, patients’ post-HD weight was measured, and BIA was measured while still in the supine position with the S10 device. After 5 min of standing, BIA was measured again using the 770 with the patient in the standing position. InBody measures a 5-point impedance, and as kHz increases, so does the impedance. We screened all patient data and excluded the ones in which this impedance was reversed. The frequency of alternating the current flowing through the body via the InBody ranges from 1 kHz to 1000 kHz. While the high-frequency currents penetrate cell membranes well and flow along the entire body water, the low-frequency currents flow mainly along the extracellular water due to the resistance of cell membranes. Therefore, the impedance is measured to be larger at the low-frequency current than at the high-frequency current [17]. The algorithm for checking impedance reversal is as follows.

1. When any one of RA, LA, TR, RL, and LL is reversed between 5 kHz and 500 kHz
2. When the impedance measured between 1 kHz and 1 MHz exceeds 50 Ω at the TR and 700 Ω at the extremities
3. When there is a sharp drop of 10 Ω or more from the TR and 100 Ω or more from the extremities between 1 kHz and 1 MHz

**Fig. 1:**
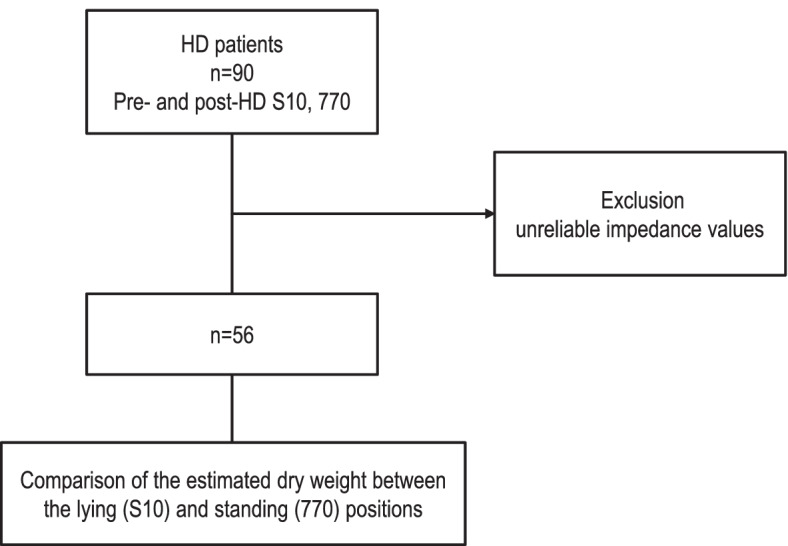
Flow chart of patients in the present study. Ninety patients were enrolled in the study and had their measurements taken with the S10 and the 770. However, a total of 56 patients were ultimately used as subjects in this study after excluding patients with unreliable impedance readings

Ninety patients were enrolled in the study and had their measurements taken with the S10 and the 770. However, a total of 56 patients were ultimately used as subjects in this study after excluding patients with unreliable impedance readings, which may have resulted from postural changes when taking readings using the 770, during which patients had to stand upright for 2 min. Patients whose results had at least one suspected error across the four measurements were excluded. The level of agreement and correlation between the S10 and the 770 for dry weight before and after HD were compared between the final 56 patients. The flow chart is presented in Fig. 1.

Actual ultrafiltration (UF) was defined as the value obtained after subtracting the post-HD weight from the pre-HD weight, while TBW changes predicted by the InBody devices were obtained by subtracting the post-HD TBW from the pre-HD TBW. Each (S10 or 770) estimated target weight, both pre- and post-HD, was considered ideal when the ECW/TBW ratio was 0.380. InBody calculates the dry weight based on the ECW to TBW ratio (ECW/TBW). In general, the ECW/TBW ratio is 0.380 because the ICW:ECW ratio is 3:2. However, in dialysis patients, the ECW/TBW increases as the amount of extracellular water increases, and the increase in extracellular water is estimated and calculated as a ratio. In other words, the dry weight of patients was calculated based on an ECW/TBW ratio of 0.380 [18, 19]. The dry weight calculation method is as follows.

$$\mathrm{Dry}\ \mathrm{weight}=\mathrm{Body}\ \mathrm{weight}-\mathrm{overhydrated}\ \mathrm{ECW}$$

$$\left(\mathrm{ECW}-\mathrm{overhydrated}\ \mathrm{ECW}\right)\div \left(\mathrm{TBW}-\mathrm{overhydrated}\ \mathrm{ECW}\right)=0.380$$

$$\mathrm{Dry}\ \mathrm{weight}=\mathrm{Body}\ \mathrm{weight}-\left(\mathrm{ECW}-0.380\times \mathrm{TBW}\right)\div \left(1-0.380\right)$$

All HD patients provided informed consent before their BIA and laboratory data were measured. The study design was approved by the Institutional Review Board of Chuncheon Sacred Heart Hospital (IRB No. 2019–07-016). This study was performed in accordance with the Declaration of Helsinki.

### Statistical analysis

Descriptive statistics are shown as mean ± standard deviation or number (%). Regression analysis was performed to demonstrate the correlation between actual weight and TBW changes with HD and to assess the correlation between the 2 BIA machines (S10 vs. 770) before and after HD. The correlation coefficient (R^2^) of regression was calculated [20]. To determine the level of agreement between the measured and the predicted UF fluid volumes, the Bland–Altman test was performed. The data were analyzed with SPSS version 23.0 (IBM Corp., Armonk, NY, USA). All reported *P*-values are two-tailed, and the statistical significance threshold was set at *P* < 0.05.

## Result

### Baseline characteristics including demographics and laboratory findings

Table 1 summarizes the demographic characteristics of the 56 patients. The average age was 54 years old, the ratio of males to females was 1:1, and the average height and weight were 161 cm and 58 kg, respectively. Thirty (53.6%) of the patients had diabetes, and 15 (26.8%) had cardiovascular disease. Their spKt/V and urea reduction rate (URR) were appropriate (spKt/V, 1.92 ± 0.36; URR, 78.84 ± 6.54). The pre-HD systolic blood pressure was 148 mmHg, and the mean diastolic BP was 72 mmHg. The post-HD systolic blood pressure was 143 mmHg, and the mean diastolic BP was 74 mmHg. Intradialytic hypotension occurred in 10 (17.9%) paitients during 4-h hemodialysis. The mean cardiothoracic ratio was 0.567 ± 0.07. The mean hemoglobin level was 11.00 ± 1.42 g/dL, and the mean serum albumin was 3.72 ± 0.32 g/dL. Other laboratory findings are described in Table 1.

**Table 1:** Baseline demographic characteristics and laboratory findings of the subjects

Variable	Subjects
	(***N*** = 56)
Sex, male, n (%)	28(50.0)
Age, year, mean ± SD	54.38 ± 12.02
Height, cm, mean ± SD	160.6 ± 9.5
Dry weight, kg, mean ± SD	58.4 ± 12.2
Diabetes, n (%)	30 (53.6)
Cardiovascular disease, n (%)	15 (26.8)
Cerebrovascular disease, n (%)	4 (7.1)
Gastrointestinal disease, n (%)	3 (5.4)
History of malignancy (cured), n (%)	6 (10.7)
Vintage, year, mean ± SD	7.26 ± 3.15
spKt/V, mean ± SD	1.92 ± 0.36
URR, %, mean ± SD	78.84 ± 6.54
SIAPR	0.243 ± 0.083
Pre SBP, mmHg, mean ± SD	148.2 ± 29.2
Pre DBP, mmHg, mean ± SD	72.0 ± 14.5
Post SBP, mmHg, mean ± SD	143.21 ± 22.08
Post DBP, mmHg, mean ± SD	74.46 ± 12.49
Intradialytic hypotension, n(%)	10 (17.9%)
CT ratio, mean ± SD	0.567 ± 0.07
Hemoglobin, g/dL, mean ± SD	11.00 ± 1.42
Albumin, g/dL, mean ± SD	3.72 ± 0.32
AST, IU/L, mean ± SD	22.43 ± 18.57
ALT, IU/L, mean ± SD	17.32 ± 12.72
BUN, mg/dL, mean ± SD	63.39 ± 17.59
Creatinine, mg/dL, mean ± SD	8.83 ± 2.81
Uric acid, mg/dL, mean ± SD	6.84 ± 1.80
Sodium, mmol/L, mean ± SD	138.27 ± 2.80
Potassium, mmol/L, mean ± SD	4.49 ± 0.63
Total CO_2_, mmol/L, mean ± SD	22.46 ± 2.70
Phosphorus, mg/dL, mean ± SD	5.10 ± 1.55
Calcium, mg/dL, mean ± SD	8.70 ± 0.69
Total cholesterol, mg/dL, mean ± SD	126.07 ± 29.12
Triglycerides, mg/dL, mean ± SD	109.15 ± 78.11
HbA1c, %, mean ± SD	6.32 ± 1.36
Transferrin saturation, %, mean ± SD	24.27 ± 10.89
TIBC, μg/dL, mean ± SD	221.86 ± 33.27
Ferritin, ng/mL, mean ± SD	244.49 ± 238.50
PTH, pg/mL, mean ± SD	249.16 ± 193.89

Abbreviations: *sp.* single pool, *URR* urea reduction rate, *SBP* systolic blood pressure, *DBP* diastolic blood pressure, *CT* cardiothoracic, *AST* aspartate aminotransferase, *ALT* alanine aminotransferase, *BUN* blood urea nitrogen, *TIBC* total iron binding capacity, *iPTH* intact parathyroid hormone

### Actual UF and TBW changes before and after HD

**Fig. 2:**
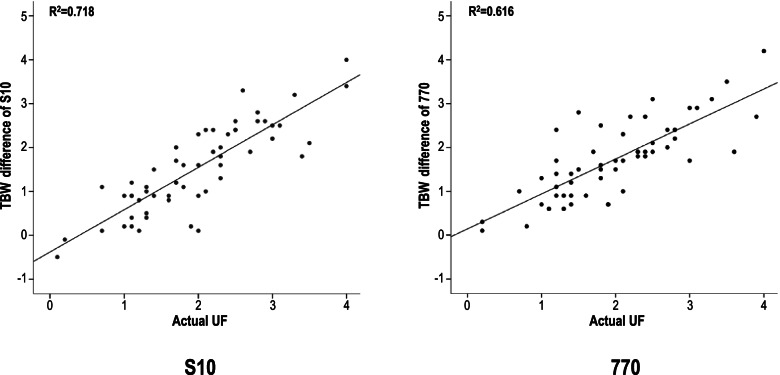
Correlations between actual ultrafiltration (UF) and pre- and post-hemodialysis differences in total body water (TBW) with patients in the lying (S10) and standing (770) positions. The R2 of TBW changes and actual UF values of the two devices were 0.718 (S10) and 0.616 (770)

**Fig. 3:**
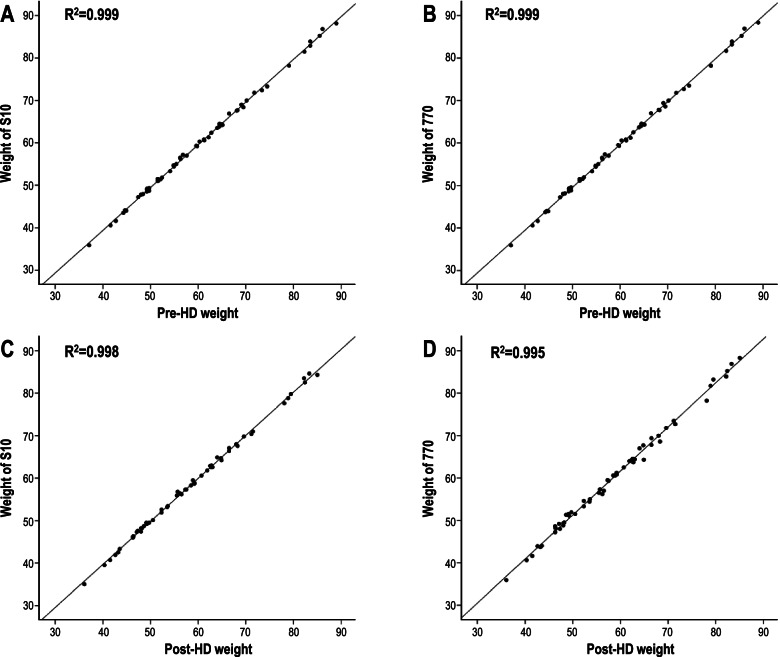
Correlations between actual weight and the estimated target weight, before and after hemodialysis (HD), with patients in the lying (S10) and standing (770) positions. Both InBody devices demonstrated almost perfect predictive power of the target weight of pre- and post- HD

In pre, and post HD, the TBW, ICW, and ECW values were well correlated between the 2 machines (Table 2). Table 3 shows the actual UF during dialysis and the UF predicted based on the pre- and post-TBW changes using the 2 InBody devices. Patients had an average of 2 kg of UF (range: 1 kg to 4 kg) during the 4 h of hemodialysis. The mean TBW change (TBW change = the difference between pre-HD TBW and post-HD TBW) measured using the InBody devices was 1.5 L (Student t-test for difference, *P* < 0.001) for the S10 and 1.7 L (*P* = 0.004) for the 770. That is, the TBW changes and actual UF values of the two devices were significantly different. The R^2^ value for their explanatory power was 0.718 and 0.616, respectively, indicating that S10 had a stronger correlation than 770 (Table 2, Fig. 2). The mean differences of actual UF and predicted TBW changes were approximately 0.4 kg for the S10 and 0.3 kg for the 770. In other words, the TBW changes measured by the InBody devices were smaller than the actual UF, although the difference between the mean readings was within 0.5 kg. In addition, regarding the pre- and post-HD estimated target weight calculated based on an ECW/TBW ratio of 0.380, the pre-HD estimated target weight was 60.0 kg for S10 and 60.1 kg for 770; there was only a difference of 0.3–0.4 kg from the pre-HD actual weight, which was 60.4 kg. However, the post-HD estimated target weight was 58.4 kg for S10 and 58.5 kg for 770, which were very close to the post-HD actual weight of 58.5 kg. Interestingly, however, the R^2^ of the pre-HD estimated target weight was 0.999 for both devices; thus, both InBody devices demonstrated almost perfect predictive power of the target weight (Fig. 3A, B). In addition, the R^2^ of the post-HD estimated target weight was 0.998 for S10 and 0.995 for 770, showing very high explanatory power (Fig. 3C, D). Supplementary Fig. 2 shows the close correlations between resistance levels (5 kHz, 50 kHz, and 500 kHz) after HD with patients in the lying (S10) and standing (770) positions.

**Table 2:** Baseline characteristics of the subjects in terms of InBody parameters

	pre		post			
**Variable (*n*** **= 56)**	**770, standing**	**S10, lying**	**770, standing**	**S10, lying**	**Pearson R**^**2a**^	**Pearson R**^**2b**^
TBW, kg, mean ± SD	32.2 ± 6.7	31.6 ± 6.8	30.4 ± 6.6	30.1 ± 6.7	0.995	0.995
ICW, kg, mean ± SD	19.4 ± 4.1	19.1 ± 4.1	18.6 ± 4.1	18.3 ± 4.2	0.995	0.994
ECW, kg, mean ± SD	12.8 ± 2.6	12.6 ± 2.6	11.9 ± 2.5	11.8 ± 2.5	0.995	0.996

*TBW* total body water, *ICW* intracellular water, *ECW* extracellular water a: correlation between pre-values, d: correlation between post-values

**Table 3:** Ultrafiltration related parameters of the subjects

Variable (n = 56)	Actual	S10, lying	770, standing	P value^**a**^	P value^**b**^	Pearson R^**2c**^	Pearson R^**2d**^
UF/TBW changes, kg, mean ± SD	2.0 ± 0.88	1.5 ± 1.0	1.7 ± 0.9	< 0.001	0.004	0.718	0.616
UF/TBW changes, kg, range	1.0 ~ 4.0	−0.5 ~ 4.0	0.1 **~** 4.2				
Pre-HD weight/estimate target weight, kg, mean ± SD	60.4 ± 12.5	60.0 ± 12.6	60.1 ± 12.6	< 0.001	< 0.001	0.999	0.999
Post-HD weight/estimate target weight, kg, mean ± SD	58.5 ± 12.1	58.4 ± 12.3	58.5 ± 12.3	0.281	0.538	0.998	0.995

*UF* ultrafiltration, *TBW* total body water, *HD* hemodialysis, TBW changes = pre-HD TBW - post-HD TBW, estimated target weight: ECW/TBW ratio = 0.385, a: difference between actual value and S10, b: difference between actual value and 770, c: correlation between actual value and S10, d: correlation between actual value and S10

### Correlation of the estimated target weight and ECW/TBW of the two Inbody machines (S10 and 770)

**Fig. 4:**
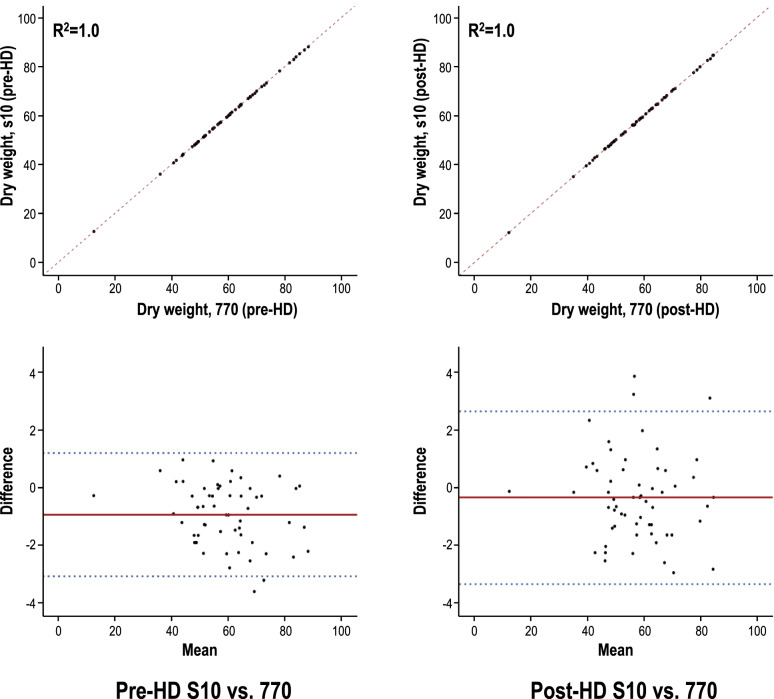
Correlations (upper) and consistency (lower) between measurements of pre-hemodialysis (HD) dry weight (left) and post-HD dry weight (right) using the two devices (S10, lying position; 770, standing position). The estimated target weight values measured by the S10 in a lying position and the 770 in a standing position were very highly correlated. In addition, most of the measured values were confirmed to be consistent in the Bland-Altman plot

As a result of evaluating the correlation and level of agreement between the two devices, it was found that estimated target weight values measured by the S10 in a lying position and the 770 in a standing position were very highly correlated, as indicated by the pre-HD and post-HD values of R^2^ = 1.000. In addition, most of the measured values were confirmed to be consistent in the Bland-Altman plot, which was used to determine the level of agreement. Therefore, this study identified that patients’ estimated target weights in the lying position using the InBody S10 device and in the standing position using the InBody 770 device in pre-HD and post-HD states were consistent (Fig. 4).

**Fig. 5:**
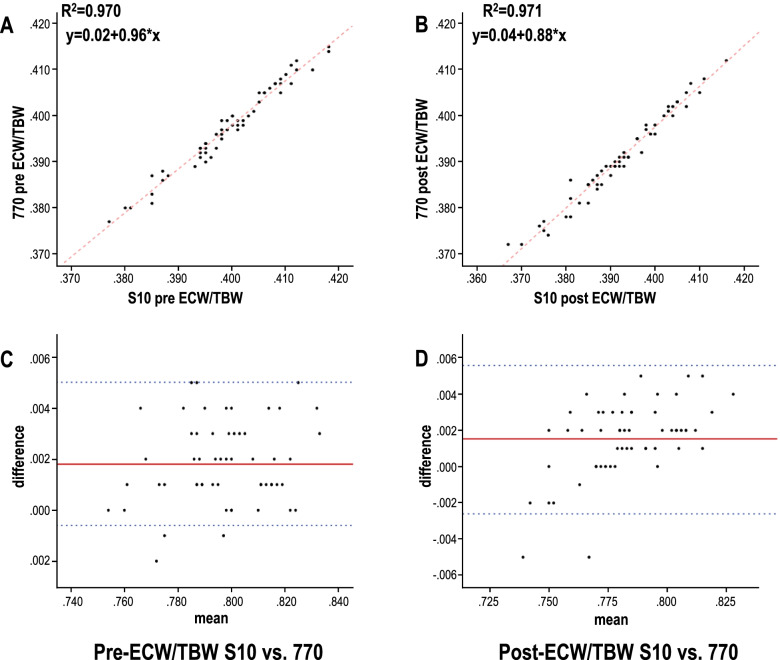
Correlations (upper) and consistency (lower) of pre- and post-hemodialysis ECW/TBW with patients in the lying (S10) and standing (770) positions. The ECW/TBW measured by the S10 in a lying position and the 770 in a standing position were very highly correlated. In addition, most of the measured values were confirmed to be consistent in the Bland-Altman plot

Meanwhile, the correlation seems perfect is that the absolute numbers for dry weight are high, we additionally analyzed the Correlation and consistency of pre- and post- ECW/TBW according to S10 and 770. It was also found that ECW/TBW measured by the S10 in a lying position and the 770 in a standing position were very highly correlated, as indicated by the pre-HD and post-HD values of *R*^2^ = 0.970 and *R*^2^ = 0.971. In addition, most of the measured values were confirmed to be consistent in the Bland-Altman plot (Fig. 5).

## Discussion

In summary, this study investigated whether the predictive power of the 770 used in a standing position is comparable to that of the S10 used in the lying position for estimating the target weight. The pre-HD and post-HD estimated target weights calculated using each device showed a very high correlation (*R*^2^ = 1.0) and significant agreement. Furthermore, the actual UF represented as TBW changes showed a high, albeit not perfect, correlation (*R*^2^ = 0.7). Therefore, according to the results of this study, even in HD patients, BIA values taken after dialysis while patients are in a standing position can be useful.

InBody calculates the amount of ECW that should be removed based on the physiological principle that the ratio of intracellular water (ICW):extracellular water (ECW) of a healthy normal person is 62:38. Thus, the ideal ECW is calculated according to the measured ICW. The overhydrated ECW is obtained from this and then subtracted from the body weight in order to calculate the dry weight. According to a recent study, ECW/TBW increases because ICW decreases with aging even without the complications accompanying edema. As a result, a formula for calculating ECW/TBW suitable for nutritional status is also used, taking ICW into account according to gender and age [19]. This formula is not recommended for use prior to dialysis because the patient has increased ECW and ICW before dialysis. However, it is recommended for evaluating the adequacy of the amount of water removed through extracellular water secretion (ECW/TBW) and dry weight after dialysis, and reflecting it in the next dialysis. Thus, if the patient remains overhydrated even after dialysis, there is a disadvantage in that an appropriate dry weight could be achieved by repeatedly performing the dry weight setting. For the diagnosis of nutritional status and sarcopenia, the muscles are overhydrated before dialysis. Therefore, evaluation should be made based on a condition close to dry weight after dialysis for the accurate diagnosis of muscle and skeletal muscle mass. Another limitation is that ECW/TBW can be affected by the degree of obesity and age.

We found that the predictive power for estimating the target weight or dry weight was comparable between the two devices. However, the estimated dry weight did not completely match the actual UF. In other words, the devices demonstrated a high correlation with regard to dry weight but failed to provide accurate values. This could be primarily attributable to the limitations of the dry weight calculation formula. InBody determines overhydration based on ECW/TBW according to the two-compartment theory. However, since subjects have increased ICW and ECW before dialysis, it is difficult to determine overhydration based only on the water ratio using ECW/(ICW + ECW = TBW). Although body composition monitors (BCMs), another type of device, are designed to use the three-compartment theory [21]—that is, measurement is performed by dividing the human body into water, fat, and lean body mass—there is insufficient evidence that BCMs have greater predictive power.

According to Kose et al. [22], the resistance of the ECW decreased instantaneously by approximately 2.3% when patients sat down, due to a shift of the interstitial and plasma fluid into the lower limbs; this shift decreased leg resistance, which constitutes a major contributor to total resistance. In their experiment, a decrease in ICW and an increase in overhydration were observed in the standing position, and the authors explained that fluid moved from ICW to ECW when patients were standing. They also stated that the steady state of the fluid can be overcome by maintaining the same posture for more than 30 min. Therefore, theoretically, in order to obtain the same value in standing and supine positions, it would be necessary to stay in one position for more than 30 min [23]. However, since we found that there was little difference between these two values after 5 min, we sought to apply this finding in clinical practice, and determined that even if a subject stood for about only 5 min, an impedance value similar to that in the supine position could be obtained.

We found that the R^2^ for the pre-HD and post-HD actual weights and the estimated dry weights was 0.999 for both devices, indicating that the predictive power of InBody devices for dry weight was quite high—to the point of being almost perfect. Nevertheless, the pre-HD dry weight was very different from the actual post-HD weight of the patients. This indicates that in pre-HD patients, both the ECW and the ICW greatly increased, so the dry weight cannot be estimated from the water ratio. However, the post-HD dry weight was very similar to the post-HD weight of the patients for both S10 and 770. In other words, patients’ post-HD weight was very close to the dry weight estimated by the InBody devices. Therefore, when estimating the dry weight using InBody, the post-HD value should be used. Furthermore, if edema remains after dialysis, the actual dry weight should be reduced according to the target weight obtained from the last InBody estimation, and then the target dry weight should be gradually adjusted through repeated InBody measurements. Additional interventional studies will be warranted to address this issue. In addition, although the pre-HD estimated target weight was very different from the actual post-HD weight, the R^2^ was very high (0.999), showing that InBody’s ability to measure body water is excellent.

In general, leg edema, serum sodium, blood pressure, chest PA’s pulmonary edema status, CT ratio, ultrasonic measurements of the inferior vena cava diameter, lungs, BNP, and the patient’s sense of well-being can be used to determine dry weight. However, when BIA is added to these clinical indicators, it will be possible to calculate a more quantified and accurate dry weight [24]. The absence of edema, which is one of the criteria used for setting the dry weight, is a very important criterion, but it is very difficult to quantify. In general, the ICF/ECF ratio is 2:1, and the fluid is in a state in which proteins and minerals are dissolved in water. ECW is approximately 98% of ECF, and ICW accounts for approximately 80% of ICF; thus, an ICW/ECW ratio of 62:38 is maintained. Regardless of the cause, increased water in tissue results in the formation of edema, which raises the ECW/TBW ratio. Dry weight is construed as the body weight without excess water, euvolemic weight, or a weight that can maintain blood pressure during dialysis [25]. In general, an ECW/TBW ratio of 0.380 is considered to reflect the absence of edema; however, in dialysis patients, in order to avoid a sudden drop in blood pressure accompanying ultrafiltration, the ideal dry weight plus a certain safe range is considered as the actual proper weight, or the practical dry weight. In addition, an adjustment of the ECW/TBW ratio is known to be necessary for patients with diabetes, hypoalbuminemia (albumin < 3.5 g/dL) [26], and heart disease. We did not apply this in the current study and defined an ECW/TBW over 0.380 as overhydration in all patients, but a Japanese study suggested that dry weight should be adjusted for HD patients with diabetes and hypoalbuminemia. For example, the authors reported that the more realistic dry weight should be 0.385 in the absence of diabetes and hypoalbuminemia; 0.395 if either one was present; and 0.405 if both were present [27]. In addition, based on clinical experience, for patients with pulmonary edema or effusion due to heart disease, even if the ECW/TBW ratio is tolerable, a stricter ECW/TBW criterion might need to be applied to avoid pulmonary congestion. In addition, as obese and muscular patients have a lower ECW/TBW than underweight patients with lower muscle mass, it is often necessary to determine the dry weight by considering these individual characteristics in the corresponding setting with reference to the existing ECW/TBW ratio. In the BIA method, as ECW represents the total of intravascular (blood) and interstitial fluid, currently there is no established method to distinguish them; however, in general, 75% of ECW represents interstitial fluid and 25% of ECW represents plasma, and it is known that the ECW measured using the BIA method is mainly due to interstitial fluid rather than intravascular fluid. However, as reported in a review by Spiegel et al. [28], many dialysis patients have excessive extracellular fluid even when their dry weight is clinically appropriate. We surmise this is because there is dehydration in the blood vessels as plasma refilling cannot keep pace with rapid ultrafiltration immediately after dialysis, whereas the interstitial fluid storage is not sufficiently corrected.

For patients with kidney failure with replacement therapy undergoing HD, maintaining a proper water ratio and nutrition in the body is a very important factor. Overhydration after HD can cause hypertension, pulmonary edema, left ventricular hypertrophy, and heart failure, which can eventually result in death from complications of cardiovascular disease. On the contrary, dehydration can cause hypotension, muscle spasm, and decreased residual renal function. Several studies using BIA have demonstrated that overhydration has an influence on mortality and morbidity in HD patients. Overhydration is defined as an excess of extracellular volume greater than 15% compared to normally hydrated people [29]. Hwang et al. reported in a systemic review and meta-analysis that overhydration increased the hazard ratio of mortality by 1.8 times [30]. Kim et al. showed that overhydration/ECW was related to mortality in dialysis patients [31]. However, Covic et al. determined that BIA-based interventions for correction of overhydration have little to no effect on all-cause mortality, although BIA improved systolic blood pressure control [32]. Kim et al. reported that monitoring volume status using BIA may help to predict all-cause death in chronic HD patients [33]. In the study by Kim et al., the InBody S10 was used, indicating that if the InBody data measured in the standing position using the 770 are similar to those of the S10, measurements taken by InBody devices can be more useful in the future.

However, there is no consensus regarding whether InBody devices can predict overhydration. In pediatric patients, BCMs predicted dry weight better than the InBody device according to a study by Yang et al. [10] In that study, UF during dialysis was evaluated by comparing pre-HD and post-HD TBW differences using Pearson correlation coefficients, and BCMs were found to be superior to InBody devices (*r* = 0.799 vs. *r* = 0.722). For BCMs, however, the sample size was small, and considering the disadvantages of BCMs, such as needing a clinician to take measurements and only producing consistent results when the lead is properly attached to the skin, it is unlikely that BCMs will be universally favorable. In addition, the results from BCMs cannot be considered more accurate because overhydration tends to be overestimated when using a BCM, which is a limitation of bioimpedance spectroscopy when used for dialysis. In clinical practice, there have been many cases when it is difficult to obtain the actual dry weight using a BCM due to its results inaccurately showing severe overhydration. However, it has been established that, since InBody devices provide estimates calculated based on the two-compartment theory, it is difficult to obtain accurate overhydration readings under conditions when one’s intracellular fluid level is increased as well.

Because of this limitation of InBody devices, it is recommended that BIA be measured to determine dry weight when the intracellular fluid level is reduced to some extent (that is, when the fluid is removed after HD.) In other words, the more euvolemic the patient is, the more stable the ICW is, meaning that dry weight can be measured more accurately. Therefore, it is expected that if dry weight continues to decrease until overhydration of the post-HD weight normalizes, dry weight can be adjusted to an appropriate level by referring to the post-HD BIA.

As a limitation of this study, there were many cases in which accurate results could not be obtained because the patient experienced difficulty standing up properly during the InBody measurements after a dialysis session. More specifically, when using the S10 device, if there is an error due to movement or other factors while measuring impedance, the person taking the measurements can immediately check it. Using the 770 device, however, it is impossible to detect whether errors have occurred during the measurement process; instead, data with errors can only be identified after the measurement is made. Measurement errors frequently occurred when the patient slightly changed his or her posture and did not maintain the same standing position for 2 min. Therefore, when using the standing position, only patients who can stand stably by themselves should be selected, and if a measurement error occurs due to the patient’s slight posture changes, it should be compensated to obtain more accurate measurement values using the 770.

In the first place, we started the current study to use the InBody 770 on dialysis patients to compensate for the InBody S10’s disadvantage of being difficult for patients to use on their own. Since the InBody 770 is a standing type, the difference between the lying and the standing positions must be minimal for it to be used on dialysis patients. However, when the InBody 770 was used, 1/3 of the patients were unable to stand upright and maintain an stationary posture for 2 min (*n* = 23), and a reversal of impedance occurred it could be also produced due to improper posture (*n* = 11). Also, unlike the electrodes of InBody S10, which is located at the ankle, the electrodes of InBody 770 were in contact with the patient’s sole, thus resulting in a very high error rate in dialysis patients with dry skin and a lot of dead skin cells. In order for us to use the InBody 770 on dialysis patients, we had to consider the following: 1) patients who can stand upright for more than 7 min (5 min for water redistribution and additional 2 min for measurement) after dialysis, 2) a system in which an alarm immediately goes off when impedance reversal occurs and remeasurement could be achieved, and 3) a material that increases the contact force between the patient’s sole and the electrode terminal. At the moment, it is possible to prevent an error by using an electrolyte tissue to wet the electrode contact area sufficiently. If an error still occurs, the electrolyte tissue can be divided into pieces and placed directly on each electrode for measurement. However, despite this current problem, it is true that the InBody 770 is more convenient to use than the InBody S10.

In conclusion, each device’s TBW prediction was not exactly accurate (*R*^2^ = 0.7), although the correlation was nonetheless very close. In addition, dry weight calculated using ECW/TBW can be measured with patients in the standing position more conveniently and autonomously using the InBody 770 device. Further research is needed to determine whether the dry weight established by periodically measuring the post-HD ECW/TBW can reduce patient mortality and morbidity.

## Supplementary Information

**Additional file 1.**

**Additional file 2.** 

**Additional file 3.** 

## Data Availability

The datasets used and/or analyzed during the current study are available from the corresponding author on reasonable request.

## Abbreviations

BIA: hioimpedance analysis
HD: hemodialysis
ICW: intracellular water
ECW: extracellular water
TBW: total body water
UF: ultrafiltration
URR: urea reduction rate
