# Supplementary material for: Consistency of the estimated target weights and ECW/TBW using BIA after hemodialysis in patients between standing and lying-down positions
Source: BMC Nephrol. 2022 Mar 17;23:106. doi: 10.1186/s12882-022-02737-3 (PMC8928688; doi:10.1186/s12882-022-02737-3)
Supplement: Supplementary file 1 — Additional file 1. [file 12882_2022_2737_MOESM1_ESM.docx]

**Supplementary table 1. The correlation of lean body mass between 770 and S10 of standing position**

| Reference Segment | InBody 720 (n=81) | |
| --- | --- | --- |
|  | R | SEE (kg) |
| Right Arm Lean | 0.998 | 0.051 |
| Left Arm Lean | 0.998 | 0.056 |
| Trunk Lean | 0.998 | 0.312 |
| Right Leg Lean | 0.996 | 0.152 |
| Left Leg Lean | 0.997 | 0.131 |

SEE: standard errors of estimate
