# Supplementary material for: Consistency of the estimated target weights and ECW/TBW using BIA after hemodialysis in patients between standing and lying-down positions
Source: BMC Nephrol. 2022 Mar 17;23:106. doi: 10.1186/s12882-022-02737-3 (PMC8928688; doi:10.1186/s12882-022-02737-3)
Supplement: Supplementary file 3 — Additional file 3. [file 12882_2022_2737_MOESM3_ESM.pptx]

## Slide 1
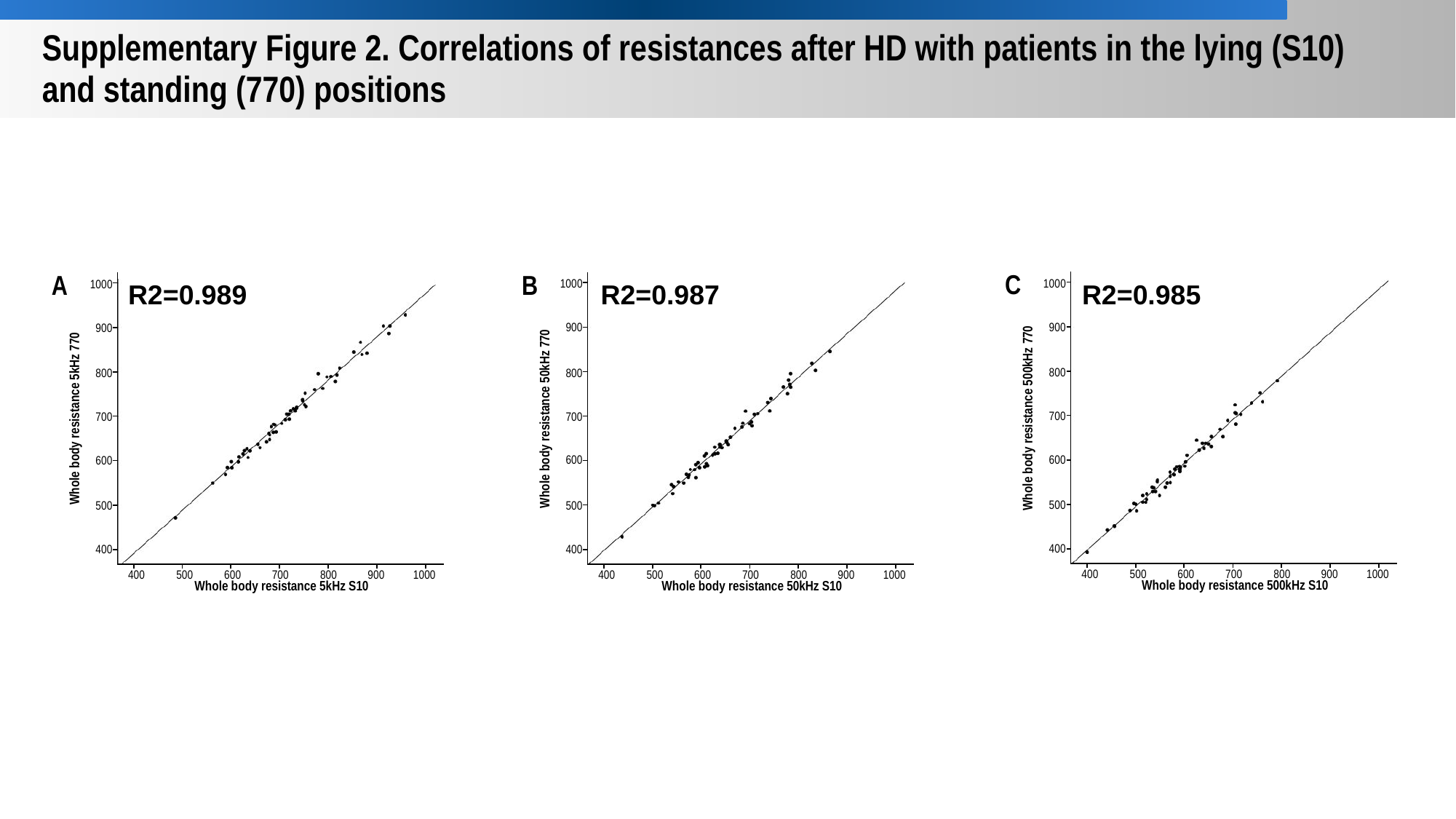

Supplementary Figure 2. Correlations of resistances after HD with patients in the lying (S10) and standing (770) positions
C
1000
900
800
700
600
500
400
Whole body resistance 500kHz 770
Whole body resistance 500kHz S10
400 500 600 700 800 900 1000
B
1000
900
800
700
600
500
400
Whole body resistance 50kHz 770
Whole body resistance 50kHz S10
400 500 600 700 800 900 1000
A
1000
900
800
700
600
500
400
Whole body resistance 5kHz 770
Whole body resistance 5kHz S10
400 500 600 700 800 900 1000
R2=0.989
R2=0.987
R2=0.985
