# Supplementary material for: Consistency of the estimated target weights and ECW/TBW using BIA after hemodialysis in patients between standing and lying-down positions
Source: BMC Nephrol. 2022 Mar 17;23:106. doi: 10.1186/s12882-022-02737-3 (PMC8928688; doi:10.1186/s12882-022-02737-3)
Supplement: Supplementary file 3 [file 12882_2022_2737_MOESM2_ESM.docx]

## Slide 1

10              15              20              25              30              35

40
35
30
25
20
15
10

Standing TR

1                    2                    3                    4                    5  

5
4
3
2
1

Supplementary Figure 1. The correlation of lean body mass between 770 and S10 of standing position

Standing RA

770 RA

5
4
3
2
1

Standing LA

770 LA

1                    2                    3                    4                    5  

770 TR

4                6                 8               10              12              14

14
12
10
8
6
4

Standing RL

770 RL

4                6                 8               10              12              14

14
12
10
8
6
4

Standing LL

770 LL

Male
Female

RA: right arm, LA: left arm, TR: trunk, RL, right leg, LL, left leg
